# Study on Factors Affecting Toric Intraocular Lens Rotation Using Intraoperative OCT—Factors Influencing IOL Deployment and Proximity to Posterior Capsule After Insertion

**DOI:** 10.3390/jcm14186599

**Published:** 2025-09-19

**Authors:** Kei Ichikawa, Seiji Tokiwa, Yoshiki Tanaka, Hiroto Toda, Yukihito Kato, Yukihiro Sakai, Kazuo Ichikawa, Naoki Yamamoto

**Affiliations:** 1 Chukyo Eye Clinic, Nagoya 456-0032, Aichi, Japan; kei@chukyogroup.jp (K.I.); stokiwa@chukyomedical.co.jp (S.T.); ytanaka@chukyomedical.co.jp (Y.T.); h-toda@chukyomedical.co.jp (H.T.); kato@chukyogroup.jp (Y.K.); sakai@chukyo-eyeclinic.jp (Y.S.); 2 General Aoyama Hospital, Toyokawa 441-0103, Aichi, Japan; 3 Center for Society-Academia Collaboration, Research Promotion Headquarters, Fujita Health University, Toyoake 470-1192, Aichi, Japan; naokiy@fujita-hu.ac.jp; 4 International Center for Cell and Gene Therapy, Research Promotion Headquarters, Fujita Health University, Toyoake 470-1192, Aichi, Japan

**Keywords:** corneal astigmatism, intraoperative optical coherence tomography (iOCT), intraocular pressure (IOP), T-IOL rotation

## Abstract

**Background/Objectives**: Cataract surgery often reveals preexisting corneal astigmatism, which can be corrected using a toric intraocular lens (T-IOL). However, postoperative T-IOL rotation may compromise correction. We investigated T-IOL rotation, focusing on deployment time and proximity to the posterior capsule (PC), using intraoperative optical coherence tomography (iOCT). **Methods**: Six different T-IOL models were inserted into acrylic simulated lens capsule models under different tacking durations (5 s, 30 s, and 60 s) and temperature conditions (23 °C, 28 °C, and 32 °C). The selection criteria for porcine lenses for examination required that they match human lens dimensions, typical of those used to train cataract surgeons. T-IOL misalignment due to vibration was assessed. Additionally, the impact of temporary intraocular pressure (IOP) reduction on T-IOL proximity to the PC was measured using iOCT in porcine eyes. **Results**: Tacking time and temperature independently affected T-IOL deployment, with shorter tacking durations and higher temperatures leading to faster deployment. Among lenses tested under identical tacking time and temperature conditions, iSert Micro Toric Aspheric 1-Piece IOL (355T3) had the slowest expansion time, while Avansee™ Preload 1-Piece Toric (YP-T3) had the fastest. Porcine eyes with a corneal white-to-white major axis < 16.0 mm fell within the 95% confidence interval for matching human lens size. Temporarily reducing IOP during surgery improved T-IOL adhesion to the PC, reducing both the occurrence and degree (from 14.0° to nearly 0°) of postoperative rotation. **Conclusions**: Optimal T-IOL deployment, temporary IOP reduction during surgery, and enhanced adhesion to the PC can reduce the risk and degree of T-IOL rotation. Intraoperative iOCT aids in monitoring T-IOL positioning, which is essential to prevent rotation. Accumulated fluid between the T-IOL and PC may contribute to rotation, which requires further investigation. These findings provide practical strategies for enhancing T-IOL stability and improving the effectiveness of astigmatism correction in cataract surgery.

## 1. Introduction

Many patients undergoing cataract surgery have preexisting corneal astigmatism. A large cohort study of over 15,000 patients found that over 36% had astigmatism ≥ 1.0 diopter (D), and an estimated 74% had ≥0.50 D [1]. One method to correct astigmatism is insertion of a toric intraocular lens (T-IOL). During cataract surgery, astigmatism can be reduced by aligning the toric axis of the T-IOL with the precalculated axis of corneal astigmatism. However, the effectiveness of such correction decreases by about 3% with each 1°of subsequent T-IOL rotation, and correction is almost eliminated by rotation of about 30° [2]. Thus, postoperative rotational stability of T-IOLs is a major concern for cataract surgeons.

The average T-IOL rotation is usually <5° [3,4]. However, depending on the T-IOL type, postoperative rotation may vary from 3% to around 20% [5]. When rotation occurs, it is usually within 14 days post-surgery, although stabilization may take up to one month [6,7].

Several factors have been reported to increase the likelihood of T-IOL rotation [8], including longer axial length [7], improper capsulotomy size or centration, incomplete removal of ophthalmic viscosurgery equipment [9], IOL unfolding time [10], changes in IOL orientation due to intraocular pressure (IOP) [9], and others. While various causes of postoperative T-IOL rotation have been reported, cataract surgeons have observed IOL deployment time varies by IOL type, temperature, viscoelastic material, and the surgical technique during routine cataract surgery. In this study, we focused on IOL deployment and adhesion between the IOL and posterior capsule (PC) as factors related to IOL rotation. Regarding factors affecting IOL deployment, we examined tacking time and temperature, measuring the time required for complete IOL deployment across various clinically used IOL types. Furthermore, we hypothesized that adhesion between the IOL and PC may be important in preventing IOL rotation. Therefore, we investigated whether IOL rotation could be suppressed by aspirating balanced salt solution (BSS) from the posterior surface of the IOL and temporarily lowering IOP to increase the adhesion area between the IOL and PC, as confirmed by intraoperative optical coherence tomography (iOCT).

## 2. Materials and Methods

### 2.1. T-IOL and Eyes

The IOLs used in this study were: TECNIS^®^ 1-Piece IOL (ZCB00, 20D, n = 27), AMO (Johnson & Johnson Surgical Vision, Inc., Irvine, CA, USA); iSert Micro Toric Aspheric 1-Piece IOL (355T3, 20D, n = 32), HOYA Corp. (Tokyo, Japan); Acrisov™ IQ Toric Single Piece IOL (SN6AT3, 20D, n = 27), Alcon Inc. (Geneva, Switzerland); TECNIS^®^ Toric Aspheric IOL (ZCT150, 20D, n = 27), AMO; Vivinex™ T-IOL (XY1A, 15.5D-26.0D, n = 27), HOYA; and Avansee™ Preload 1-Piece Toric (YP-T3, 20D, n = 27), Kowa Company, Ltd. (Aichi, Japan).

Fresh porcine eyes (aged about 5–6 months) from a local slaughterhouse were used. The eyes were stored at 4 °C with eyelids closed to prevent damage and minimize air exposure, and were used within 10 h post-enucleation. Due to their morphological similarity to human eyes, porcine eyes are frequently used as ex vivo animal models in vision science research [11,12,13]. A total of 40 porcine eyes were prepared for the experiments.

This study adhered to the ARVO Statement for the Use of Animals in Ophthalmic and Vision Research. Ethics committee approval from Chukyo Eye Clinic (Nagoya, Aichi, Japan) was ruled unnecessary since the porcine eyes were byproducts of the slaughter process and not harvested for this study.

To investigate postoperative T-IOL rotation, patients who underwent cataract surgery with IOL (355T3) insertion were examined for IOL rotation within 1-week postoperatively. Approval for this clinical study was obtained from Chukyo Eye Clinic ethics committee (reception number: 20220817-01).

### 2.2. Equipment

To standardize the continuous curvilinear capsulorhexis (CCC) size in porcine eye lenses, a ZEPTO^®^ device was used. All experiments were conducted using an active-fluidics and torsional phacoemulsification device (CENTURION^®^ VISION SYSTEM, Alcon Laboratories, Inc., Vernier-Geneva, Switzerland) by one ophthalmologist experienced in cataract surgery at Chukyo Eye Clinic.

iOCT measurements were performed immediately after T-IOL insertion using ZEISS ARTEVO800. iOCT imaging was performed using either ARTEVO 800’s built-in iOCT functionality or the ReScan 700 module. IOP in porcine eyes was monitored using a veterinary-grade tonometer, Tono-Pen AVIA (Reichert, Inc., Depew, NY, USA).

### 2.3. Experimental Procedure

#### 2.3.1. Effects of Tacking Time and Temperature on IOL Deployment

**Figure 1:**
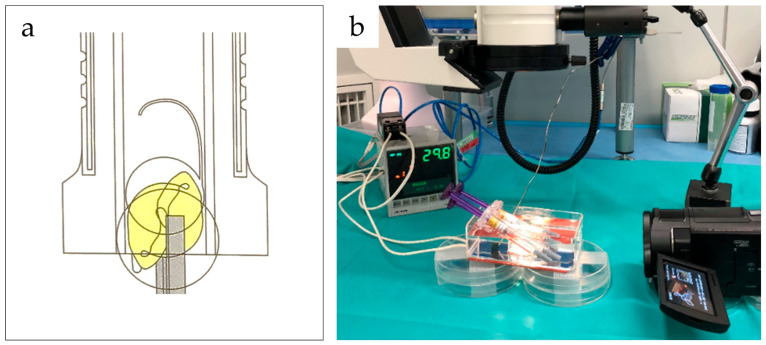
(**a**) Conceptual diagram illustrating the tacking state of the IOL folded and stored in the cartridge. (**b**) Experimental setup, including a device for maintaining the temperature of experimental materials, a surgical microscope, and a video recorder.

During microincisional IOL insertion, an injector was employed. The IOL was folded once within the injector prior to insertion (Figure 1a). As some injector instructions recommend tacking times (i.e., the duration the IOL remains folded within the injector before insertion) of no more than 5 min, we measured the time required for full IOL deployment (complete unfolding and positioning of the IOL within the capsular bag during cataract surgery) under various tacking durations (5 s, 30 s, and 60 s).

We also examined temperature effects on IOL deployment. Oxyglutathione BSS (BSS, Santen) was placed in a water tank containing an acrylic simulated lens capsule model at room temperature. The acrylic artificial lens capsule model had a diameter of approx. 1 cm, with an anterior capsule incision size of approx. 5 mm (Supplementary Videos S1–S3). A viscoelastic agent (Subenil, Chugai) was used and maintained at three experimental temperatures: 23 °C, 28 °C, and 32 °C. A photograph of the experimental setup is shown in Figure 1b. Side-view videos of the tank were recorded to capture the unfolding process, and time to complete IOL deployment was measured.

After equilibrating all components to the designated temperatures, tacking was performed for 5 s, 30 s, and 60 s, respectively. Each IOL was then injected into the acrylic simulated lens capsule model. Timing began when the trailing haptic (rear support portion) made contact with the acrylic simulated lens capsule model. IOL deployment was monitored through an operating microscope. Deployment was considered complete when the haptics engaged the equator of the pseudo-lens, stopped changing shape, and the optic portion fully flattened.

#### 2.3.2. Size Comparison Between Porcine and Human Lenses

We aimed to select porcine eye lenses that closely resemble human lens dimensions for research purposes and for training junior doctors in cataract surgery. To this end, we investigated whether the porcine lens diameter could be predicted from its corneal diameter. We measured the major and minor corneal diameters, referred to as white-to-white (WTW), in porcine eyes. We then measured the corresponding major and minor axes of the lens while it remained within the eyeball. For comparison, we performed similar measurements on human eyes, recording both corneal and lens diameters (major and minor axes) using a measuring microscope (STM6, Olympus) equipped with a 3× magnification objective lens.

#### 2.3.3. Assessment of T-IOL Misalignment Due to Vibration

Porcine eyes (n = 5) with lens dimensions similar to those of human lenses were selected, and a CCC was created using a ZEPTO^®^ device to ensure consistency. The crystalline lens contents were removed, and the 355T3 IOL, which demonstrated the slowest deployment, was inserted into the capsular bag. The eye was inflated with BSS, the incision was sutured with 9-0 nylon (1 stitch), and the viscoelastic substance was removed by irrigation-aspiration. The positions of the IOL and PC were confirmed by iOCT. Within 90 s of lens insertion, porcine eyes were removed from the fixed table and subjected to lateral and vertical shaking (5 cycles at 1 cycle/s), followed by vigorous vertical vibration (10 cycles at 2 cycles/s) with a travel distance of 20 cm. This vibration protocol was designed to simulate human head movements and the physical activity of standing from a seated position.

Next, the IOL was returned to its original position, and IOP was adjusted by removing BSS trapped between the IOL posterior surface and the PC. IOP was confirmed to be within 20 mmHg using a Tono-Pen to simulate physiological conditions of the human eye. The positions of the IOL and PC were reassessed by iOCT (by this time, the IOL was fully deployed). Similarly, the eyeball was subjected to total of 20 vibration cycles, and iOCT was used to evaluate toric IOL alignment and PC status.

### 2.4. IOL Rotation Within One Week Postoperatively

To investigate postoperative T-IOL rotation, patients (24 eyes, age: 75.5 ± 9.9 yrs.) who underwent cataract surgery with IOL (355T3) insertion were examined for IOL rotation within one week postoperatively. Surgery was completed after carefully observing the required time for complete IOL expansion. As usual practice, 8 randomly selected eyes underwent surgery with viscoelastic substance aspiration to complete the procedure (Group A). The remaining 16 eyes underwent surgery with viscoelastic substance aspiration first, followed by aspiration of BSS from the posterior IOL surface to temporarily lower IOP and enhance lens adhesion to the posterior capsule (Group B).

### 2.5. Statistical Analysis

Data are presented as mean ± standard deviation (SD). The Kruskal–Wallis test, followed by Scheffe’s post hoc test, was used to compare three or more independent groups. Friedman’s test was applied for comparisons among related groups. The relationship between immediate postoperative IOP and axial deviation was examined using Spearman’s rank correlation coefficient. SPSS Statistics 24 (IBM Corp., New York, NY, USA) was employed with *p* < 0.05 deemed significant.

## 3. Results

### 3.1. Factors Influencing T-IOL Rotation: IOL Deployment

**Figure 2:**
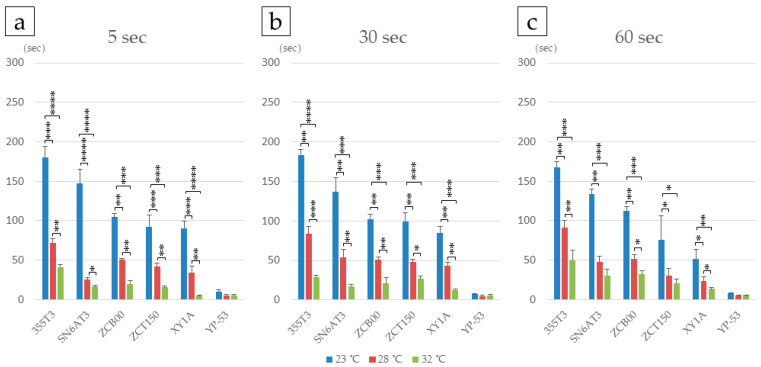
Effect of tacking time and temperature on T-IOL deployment time by IOL type. Deployment times are shown for tacking durations of 5 s (**a**), 30 s (**b**), and 60 s (**c**) at three temperatures: 23 °C, 28 °C, and 32 °C for six IOL types. *: *p* < 0.05, **: *p* < 0.01, ***: *p* < 0.005, ****: *p* < 0.001.

The time required for complete IOL expansion (deployment time) varied depending on the tacking duration within the cartridge and the temperature of the surgical environment and materials. Shorter tacking durations and higher temperatures resulted in shorter IOL deployment times. For a given tacking time, IOLs expanded more quickly at higher temperatures. Among all tested IOLs, the YP-53 consistently exhibited the fastest deployment across all temperature and tacking time conditions, whereas the 355T3 showed the slowest deployment at 23 °C, except when tacked for 60 s (Figure 2, Table 1 and Supplementary Videos S1–S3).

These findings demonstrate that different IOL models require varying deployment times to achieve complete expansion under identical tacking and temperature conditions. Ophthalmic surgeons should be aware that deployment time is IOL-specific and may affect surgical workflow and patient outcomes.

### 3.2. Factors Affecting T-IOL Rotation: Lens Size

**Figure 3:**
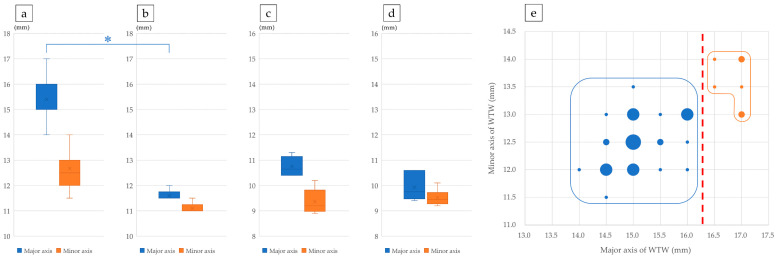
Comparison of corneal white-to-white (WTW) and lens dimensions between porcine and human eyes. (**a**) Major and minor diameters of porcine corneal WTW. (**b**) Major and minor diameters of human corneal WTW. (**c**) Major and minor diameters of porcine lenses. (**d**) Major and minor axes of human lenses. (**e**) Distribution of porcine corneal WTW measurements relative to the mean ± 2SD of human lens dimensions. Blue circles indicate porcine eyes with WTW smaller than or within the human lens range; orange circles indicate WTW greater than the human lens range. Circle size reflects the number of observations. *: *p* < 0.001.

To investigate factors contributing to T-IOL rotation, iOCT measurements were conducted using a ZEISS ARTEVO800 immediately after T-IOL insertion. Subsequently, we conducted IOL rotation experiments using porcine eyes. First, we established selection criteria for porcine lenses that closely resemble human lenses in size. The corneal WTW diameter and lens dimensions of both porcine and human cadaveric eyes were measured. The major axis of porcine corneal WTW was significantly longer than that of human corneal WTW (*p* < 0.05). Although no significant difference was found in the minor axis, porcine corneal WTW tended to be longer (Figure 3a,b). For both the major and minor lens diameters, there were no statistically significant differences between porcine and human lenses, though porcine lenses tended to have larger major axes and smaller minor axes (Figure 3c,d). We then calculated the estimated ranges for human lens dimensions based on the mean ± 2SD: the major axis ranged from 8.96 to 10.91 mm, and the minor axis from 8.93 to 10.10 mm. By plotting porcine corneal WTW values against these human lens dimensions, we found that a porcine corneal WTW ≥ 16.5 mm typically corresponds to a porcine lens larger than the human lens (Figure 3e). Based on these findings, porcine lenses within the human size range were selected for use in this study.

### 3.3. Factors Influencing T-IOL Rotation: Proximity of Porcine Posterior Lens Capsule to IOL

**Figure 4:**
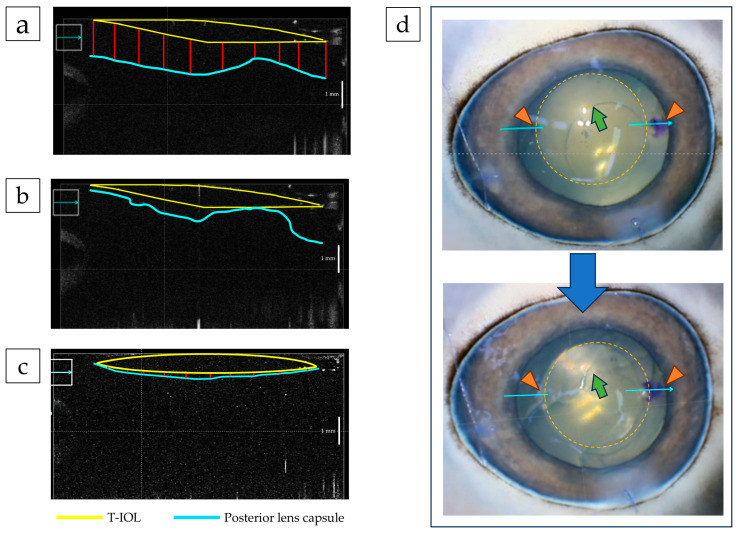
Positional relationship between the T-IOL and PC in porcine eyes, visualized by iOCT. (**a**) iOCT image at high IOP, before aspiration of BSS from the IOL-PC gap. The red line indicates the IOL-PC distance. (**b**) iOCT image taken during temporary IOP reduction while BSS was being aspirated from the IOL-PC gap. (**c**) iOCT image after BSS aspiration, showing the final T-IOL and PC configuration with the red line indicating the IOL-PC distance. (**d**) T-IOL position before and after ocular vibration following temporary IOP reduction. The T-IOL position remained unchanged after vibration (orange arrowheads). The yellow dotted line outlines the T-IOL: the green arrow indicates the anterior capsulotomy edge.

In five porcine eyes, T-IOLs were implanted using standard surgical procedures under high IOP after removal of the crystalline lens contents. The positions of the IOL and PC were confirmed by iOCT (Figure 4a). After approx. 90 s at 23 °C, during which the IOL had not yet fully unfolded, vigorous ocular vibration induced a mean IOL eccentricity angle of 14.0° ± 3.7° (20° in one eye, 15° in two eyes, and 10° in two eyes). Next, after allowing the IOL to fully expand, BSS was aspirated from the IOL-PC interface, intentionally lowering the IOP to ≤20 mmHg to increase IOL-capsule proximity (Figure 4b). When the same vibration protocol was repeated, no IOL eccentricity was observed in any of the eyes (eccentricity angle ≈ 0°; Figure 4c). iOCT imaging revealed a further reduction in the gap between the IOL and PC, suggesting an increased contact area (Figure 4d). The IOL–PC distance was measured at 10 randomly selected points per eye. The mean distance across the five eyes was 1.05 mm without IOP reduction (Figure 4a), which decreased to 0.07 mm with IOP reduction (Figure 4c, *p* < 0.001).

### 3.4. Suppression of IOL Eccentricity by Temporary Low IOP

Patients (24 eyes, age: 75.5 ± 9.9 yrs.) who underwent cataract surgery with implantation of IOL (355T3) were examined for IOL rotation within one week postoperatively. In Group A (8 eyes; age: 74.9 ± 9.6 yrs.) surgery was completed by aspirating the viscoelastic material as per standard surgery. The mean IOP was 13.2 ± 2.5 mmHg and the mean lens eccentricity was 2.6° ± 2.2°. In Group B (16 eyes: age: 76.6 ± 10.1 yrs.) the procedure included additional aspiration of viscoelastic material from the IOL-PC gap, a temporary reduction in IOP, and iOCT confirmation of IOL contact with the PC before surgery completion. The mean IOP in this group was 8.4 ± 1.5 mmHg, and no IOL eccentricity was observed (Supplementary Figure S1). These findings demonstrate that full expansion of the IOL with sufficient BSS aspiration to temporarily lower IOP can suppress postoperative rotation, achieving an eccentricity angle of 0°.

## 4. Discussion

Postoperative rotation of T-IOLs remains a global challenge for cataract surgeons, with several contributing factors identified [8]. During routine cataract procedures, I observed variability in the time required for full deployment across different models. This study investigated whether the immediate postoperative relationship between the T-IOL and the PC influences rotation, using iOCT to identify potential new factors.

First, we explored variables affecting T-IOL deployment. With the increasing prevalence of microincision cataract surgery, injectors have become standard for inserting IOLs. Tacking duration, which is the amount of time an IOL is held in the injector, was found to affect deployment time. Longer tacking times led to slower unfolding of the IOL. However, the extent of this effect varied by IOL type and surgical practice. For example, at 23 °C, deployment time was relatively stable for models ZCB00, ZCT150, and XY1A across different tacking times. In contrast, the 355T3 showed slower deployment at longer tacking durations, particularly at 30 s and 60 s. The SN6AT3 also showed slower deployment regardless of tacking duration. Among all lenses tested, the YP-T3 consistently unfolded the fastest under all conditions.

Rapid deployment of hydrophobic acrylic IOLs is clinically advantageous [14,15]. It was reported that increased temperature can reduce unfolding time [10,16]. It is common practice to store ophthalmic viscosurgical devices in a cool place with light shielding. Product inserts recommend allowing them to reach room temperature at least 30 min before use. In silico simulation analysis estimates the temperature of the human lens in vivo to range from 35.0 °C to 37.5 °C [17,18]. In our experiment, porcine eyes were maintained at 36 °C, but during surgery, the intraocular temperature temporarily dropped to 28 °C when the BSS reached the PC during surgery, and the intraocular temperature was approx. 32 °C when the IOL was injected [10]. Therefore, in this study, we measured IOL deployment time at three temperature points: 23 °C and 32 °C at room temperature, and 28 °C as an intermediate temperature. Our findings revealed time required for full deployment decreased as temperature increased to 23 °C, 28 °C, and 32 °C for all T-IOLs. Thus, this study highlights the significance of using T-IOLs within the tested temperature range, as it affects time for complete deployment.

The results of this study revealed IOL deployment was more affected by temperature than tacking time. One reason for this is the material of IOLs becomes hard (glass-like) at low temperatures and soft (rubber-like) at high temperatures. The temperature at which this transition occurs is called the glass transition temperature [19]. The glass transition temperature of IOLs used in this study has not been disclosed by manufacturers, so details are unclear. However, since the materials and synthesis methods vary among manufacturers, it is presumed that glass transition temperatures differ, resulting in variations in lens deployment due to temperature differences.

In our investigation of T-IOL rotation using porcine eye lenses, we observed variations in the sizes of porcine crystalline lenses compared to human lenses. While many porcine lenses are similar in size to human lenses, some are slightly larger. To ensure our research using T-IOLs intended for human cataract surgery was relevant, we aimed to match the size of porcine lenses used with that of human lenses and discovered a correlation between the size of the porcine lens and WTW measurement of the cornea. Although previous attempts to measure porcine eye structures using MRI lacked sufficient resolution for accuracy [20], our study yielded significant findings. We determined the size of a porcine lens falling within the mean ± 2 SD of the major and minor axes of a human lens, could be predicted based on its WTW measurement. Porcine eyes with a corneal major axis < 16.0 mm were most likely to have lens sizes equivalent to the human lens.

Based on our findings, we adjusted the laboratory room temperature to 23 °C and ensured that the ophthalmic viscosurgical device or BSS used for manipulating the T-IOL was warmed to room temperature, consistent with the environment. The T-IOL was inserted with the lens interior aspirated, focusing on the 355T3, which demonstrated the slowest deployment among tested lenses and the highest rotation risk [21,22,23]. Furthermore, the rationale for selecting 355T3 was its prolonged deployment time, suggesting successful rotation control with this lens might extend to other models. In cataract surgery, the time required for complete deployment varies by IOL type, temperature, and surgical technique. At our hospital, surgery usually concludes approx. 90 s after IOL insertion. Since the patient’s head position changes from supine to sitting, standing, and walking during this time, it is possible that cataract surgery may conclude before complete deployment under certain conditions. Accordingly, we conducted a vibration experiment in porcine eyes to simulate head position changes under the condition where complete IOL deployment is the most delayed (IOL: 355T3, temperature 23 °C). We observed an IOL-PC gap of approx. 1 mm before lowering IOP, which facilitates IOL rotation upon eye movement. This scenario resembles aquaplaning, where a water film forms between the tires of a car and the road surface, causing it to float rendering the steering wheel and brakes ineffective. We conducted a simple experiment with the T-IOL suspended in a BSS-filled container. When the container was tilted to temporarily displace BSS between the T-IOL and the container, upon restoring the BSS to the periphery of the T-IOL, no movement was observed. This lack of movement is attributed to the constant contact between the T-IOL and the container. Furthermore, even if the T-IOL suspended in the BSS was pressed against the container briefly, it remained stationary (Supplementary Video S4). In a study using a simulated lens, it was observed that higher temperatures of and warming of the IOL led to increased adhesion between the simulated lens and the IOL. At physiological temperatures, acrylic foldable IOLs demonstrated the highest adhesion to the PC, followed by silicone IOLs and PMMA IOLs [23]. Acrylic foldable IOLs have also been reported to show lower hydrophobicity and surface roughness compared to silicone IOLs and PMMA IOLs [24]. In actual surgery, the time for IOL deployment at 28 °C is most suitable for operability, because if an IOL deploys in too short a time, it is challenging to fine-tune its position. Conversely, surgeons should be aware that IOL deployment times vary widely by IOL type at 23 °C. BSS between the T-IOL and PC was intentionally aspirated, resulting in a slight decrease in IOP. iOCT revealed the IOL-PC gap narrowed and contact between the T-IOL and PC was observed at some points. Even when the eyeball was vibrated in this state, the T-IOL did not rotate.

If T-IOL rotation is <10°, eye refraction change is <0.50 D. Consequently, minor rotation along the small axis does not hinder satisfactory astigmatism correction with T-IOLs [25]. However, it is important for patient quality of vision to prevent T-IOL rotation as much as possible.

Limitations of this study include examination of T-IOL deployment temperature at only three points: 28 °C, 32 °C, and 23 °C, representing typical operating room conditions. Similarly, tacking times were only investigated for 5 s and 60 s, with 30 s being the norm in routine surgery. Furthermore, not all types of T-IOLs were studied, and verification was conducted with only the 355T3, which is known to be the most prone to rotation. In vibration experiments using porcine eyes, experiments were not conducted under different IOP conditions with the IOL complete deployment. Another limitation of our porcine eye experiments is that we did not test vibration after fluid aspiration but before full IOL expansion, which would help isolate the specific contribution of IOL-PC proximity vs. complete deployment in preventing rotation. These findings suggest that surgeons should allow adequate time for complete IOL deployment before concluding surgery, particularly when using slower-deploying IOL models like the 355T3 and consider the benefits of temporarily reducing IOP through BSS aspiration to enhance IOL-PC adhesion.

## 5. Conclusions

In a wet lab utilizing porcine eyes handled by cataract surgeons in training, porcine eyes matching the size of the human lens, with WTW < 16.0 mm, were used. To minimize T-IOL rotation, it is recommended to wait until the T-IOL is fully deployed. Adjusting IOP to expel BSS from between the T-IOL and the PC, and increasing the area of adhesion surface between them, can decrease T-IOL rotation. Employing iOCT to monitor the PC condition and surface of the T-IOL is beneficial in suppressing T-IOL rotation. Fluid between the T-IOL and PC was identified as a factor contributing to intraocular lens rotation.

## Figures and Tables

**Table 1:** IOL deployment time under each experimental condition. Values indicate mean deployment time (maximum time/minimum time).

Lens	Tacking Duration	23 °C	28 °C	32 °C
355T3	5 s	180.2 (200.0/166.4)	72.2 (76.0/66.0)	41.1 (44.9/38.5)
	30 s	183.4 (190.2/173.0)	83.4 (96.0/71.6)	28.5 (32.2/24.7)
	60 s	167.8 (174.6/161.0)	91.3 (100.2/78.8)	50.0 (65.9/34.9)
SN6AT3	5 s	147.2 (165.0/129.4)	25.2 (29.0/23.0)	16.6 (17.5/15.1)
	30 s	136.7 (150.1/118.3)	54.3 (67.4/47.2)	17.6 (19.5/15.6)
	60 s	133.6 (140.0/127.2)	47.5 (54.5/37.9)	30.4 (37.6/19.2)
ZCB00	5 s	104.3 (110.0/98.0)	50.3 (53.0/48.0)	20.0 (25.0/15.1)
	30 s	102.3 (110.2/95.0)	51.0 (55.4/48.2)	21.0 (29.6/14.4)
	60 s	112.7 (120.0/108.1)	51.7 (55.0/45.2)	31.7 (35.3/25.0)
ZCT150	5 s	92.3 (112.2/77.0)	41.4 (50.5/37.2)	15.3 (18.1/14.0)
	30 s	99.7 (113.0/86.2)	48.0 (53.1/42.3)	26.7 (34.0/21.9)
	60 s	75.8 (116.5/65.2)	29.7 (42.3/18.2)	20.9 (27.3/16.5)
XY1A	5 s	90.3 (101.5/80.4)	34.5 (45.3/25.0)	5.0 (6.0/4.0)
	30 s	84.7 (96.1/78.5)	43.0 (50.0/37.9)	12.7 (14.0/10.9)
	60 s	51.7 (67.6/38.8)	23.4 (30.9/18.2)	13.5 (16.2/11.5)
YP-53	5 s	9.8 (11.6/6.1)	5.4 (6.7/4.6)	4.7 (6.8/3.3)
	30 s	7.1 (8.7/6.2)	5.1 (6.6/3.9)	5.7 (7.5/4.3)
	60 s	7.6 (8.7/7.0)	4.9 (5.7/3.7)	5.6 (5.9/5.1)

## Data Availability

Data is contained within the article or Supplementary Materials. Further inquiries can be directed to the corresponding author.

## Supplementary Materials

The following supporting information can be downloaded at https://www.mdpi.com/article/10.3390/jcm14186599/s1. Video S1: Video of SN6AT3 unfolding at 23 °C. (**a**) Tacking 5 s. (**b**) Tacking 30 s. (**c**) Tacking 60 s, Video S2: Video of SN6AT3 unfolding at 28 °C for tacking times. (**a**) 5 s, (**b**) 30 s, and (**c**) 60 s, Video S3: Video of SN6AT3 unfolding at 32 °C, for tacking times. (**a**) 5 s, (**b**) 30 s, and (**c**) 60 s, Video S4: Seal the T-IOL in a container of BSS and shake it. If BSS between the T-IOL and container wall was temporarily vacant, the T-IOL remained fixed when the container was subsequently shaken, because the T-IOL adhered to the container wall. Furthermore, the position of the T-IOL suspended in the BSS was fixed by lightly pressing it against the wall of the container. Supplementary Figure S1: Relationship between IOP and IOL eccentricity in Groups A and B. Correlation was found between IOP and IOL eccentricity (Spearman’s rho; *p* < 0.05).
