# Supplementary figures and images for: Study on Factors Affecting Toric Intraocular Lens Rotation Using Intraoperative OCT—Factors Influencing IOL Deployment and Proximity to Posterior Capsule After Insertion
Source: J Clin Med. 2025 Sep 19;14(18):6599. doi: 10.3390/jcm14186599 (PMC12470799; doi:10.3390/jcm14186599)

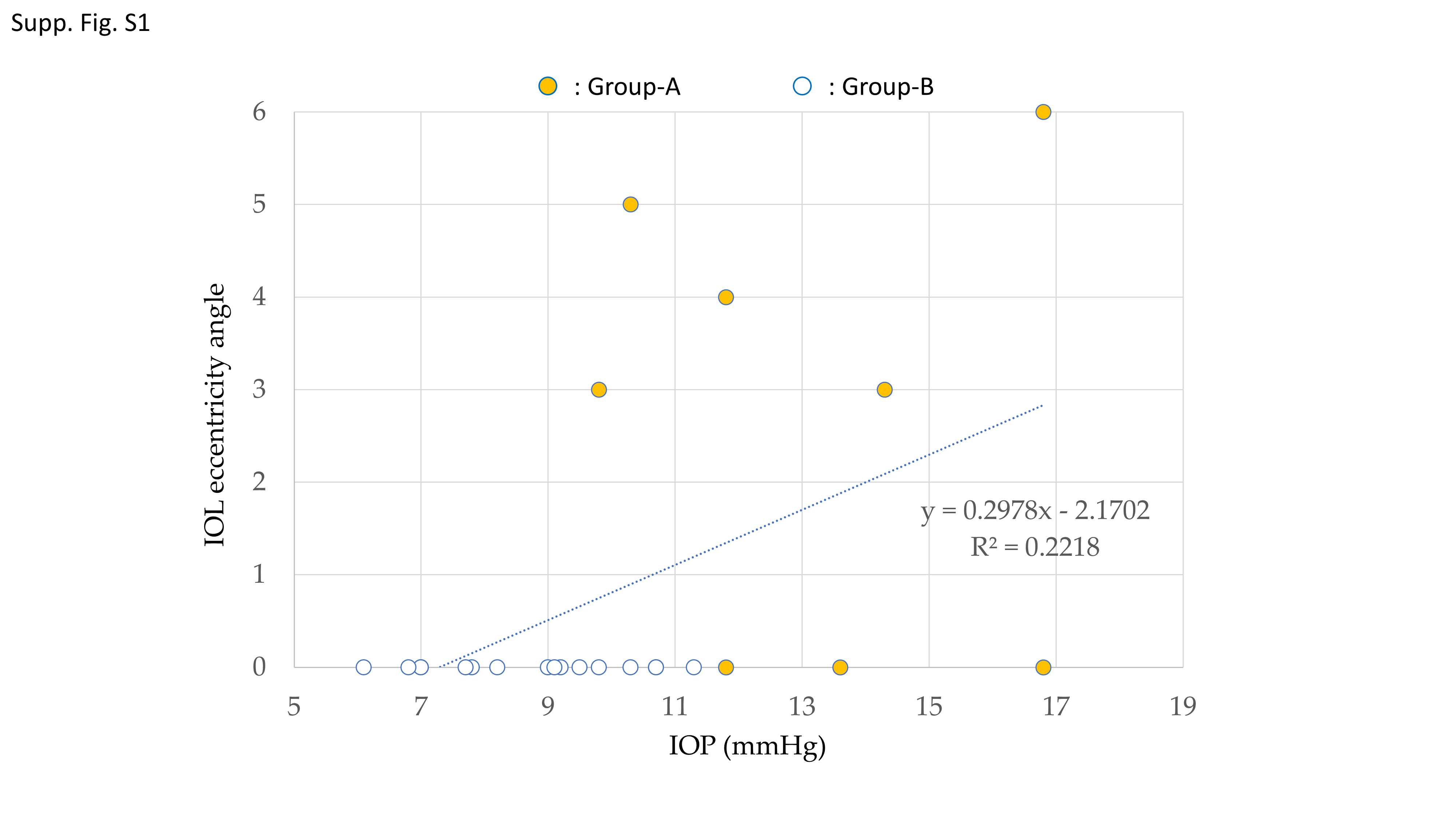

Supplement: Supplementary file 1 [file jcm-14-06599-s001.zip › JCM-3674028-Supplementary Materials/Suppl Figure S1.TIF]
